# Information-optimal local features automatically attract covert and overt attention

**DOI:** 10.1038/s41598-022-14262-2

**Published:** 2022-06-15

**Authors:** Serena Castellotti, Anna Montagnini, Maria Michela Del Viva

**Affiliations:** 1 Department of Neurofarba, University of Florence, Florence, Italy; 2 Institut de Neurosciences de la Timone, CNRS and Aix-Marseille Universitè, Marseilles, France

**Keywords:** Attention, Perception

## Abstract

In fast vision, local spatial properties of the visual scene can automatically capture the observer’s attention. We used specific local features, predicted by a constrained maximum-entropy model to be optimal information-carriers, as candidate “salient features''. Previous studies showed that participants choose these *optimal* features as “more salient” if explicitly asked. Here, we investigated the implicit saliency of these *optimal* features in two attentional tasks. In a covert-attention experiment, we measured the luminance-contrast threshold for discriminating the orientation of a peripheral gabor. In a gaze-orienting experiment, we analyzed latency and direction of saccades towards a peripheral target. In both tasks, two brief peripheral cues, differing in saliency according to the model, preceded the target, presented on the same (valid trials) or the opposite side (invalid trials) of the *optimal* cue. Results showed reduced contrast thresholds, saccadic latencies, and direction errors in valid trials, and the opposite in invalid trials, compared to baseline values obtained with equally salient cues. Also, *optimal* features triggered more anticipatory saccades. Similar effects emerged in a luminance-control condition. Overall, in fast vision, *optimal* features automatically attract covert and overt attention, suggesting that saliency is determined by information maximization criteria coupled with computational limitations.

## Introduction

Visual attention is used to prioritize significant objects in a complex visual environment^1–4^. Selective visual processes can be set in place automatically and very quickly, as proven by the intrinsic saliency of some visual stimuli which obtain priority processing (exogenous attention). A salient stimulus automatically pops out of a visual scene, suggesting that saliency is computed pre-attentively across the entire visual field. This bottom-up saliency is largely independent of the nature of the specific task at hand, it operates very rapidly and is primarily driven by the nature of the stimuli, although it can be influenced by contextual effects of the visual surroundings (e.g., figure–ground). Other types of attentional selective processes can be driven in a top-down manner and influenced by task-dependent cues requiring a voluntary ‘effort’ and cognitive strategic processes (endogenous attention); for example, an instruction like ‘look for the red horizontal target’. Both mechanisms can operate in parallel, although their characteristic time courses are different (for a review, see^5^).

The principles driving the bottom-up saliency of visual features are still subject of intense debate. Phenomenologically, the saliency of a visual stimulus depends on several physical properties (e.g., luminance, color, orientation of contours, edges) and it scales with the degree of dissimilarity of each property with respect to the statistics of that property in the surround (e.g., the stimulus luminance vs. the background luminance)^6–8^. However, a stimulus’ saliency can often also be appreciated with isolated stimuli.

Most models proposed for the estimation of the bottom-up saliency map rely on the empirical observation of neurons’ biological properties and generally define a-priori the dimensions along which a stimulus’ saliency can vary (luminance, edge orientation, etc.). Some approaches compute the local maxima of the contrast of these dimensions with respect to the surround to identify salient features^6,8^. Eye tracking models, on the other end, have tried to relate visual saliency to the locations where fixations occur^9–11^. In fact, in the presence of multiple information cues in a complex natural scene, the pattern of ocular fixations is often used as an operational definition of the saliency map of the scene^12^.

Here, instead of considering the saliency map as consisting of known elements as addressed in previous studies (luminance, color, etc.), we adopt a different point of view following a recent approach for efficient extraction of visual features^13^. These authors provided a definition of saliency by starting from a general problem faced by the visual system: the extraction of biologically-relevant information from a large flux of input data in the shortest possible time, upon which survival depends^14^. In these conditions, as already suggested by many, an early and intensive data reduction must be operated^15–17^, leading to a compact representation of the visual scene (“primal sketch”)^18,19^. Indeed there are physical reasons beneath the need for early data reduction: processing is limited by the energy costs of neural activity^20–22^, the number of neurons, and the limited time available for the task. However, in these previous studies, the features composing the sketch were based on few simple primitives (edges, bars), and were defined a priori based on known properties of receptive fields and the edge detection ability of the visual system^18,19^. Instead, the aforementioned approach^13^ considers computational limitations as a central element together with the information optimization principle to extract a number of visual features from the statistics of natural images, composing the compact saliency map^13^. Specifically, this approach aims to model an early visual stage, operating in fast vision, acting as a filter that retains only a very limited number of image features for further processing. To define “fast vision” we use the operational definition derived from Thorpe’s work^23^. For highly demanding tasks fast vision processing is considered to occur within delays below 150 ms in higher visual areas, and as low as 25–50 ms for early visual areas processing^24^. The features selected are those that transmit in output the maximal information allowed by the system constraints of this early-stage filter (*constrained maximum-entropy* model). Very general constraints were used, related to the energy costs determining the need for compression mentioned above. The model assumes that 1) the number of neurons composing this stage is limited, therefore this system can recognize in its input only a fixed number of pre-determined visual features (limited storage occupancy); 2) there is a tight upper bound on the total amount of data that can be transmitted as output (limited bandwidth). Such a system is optimal from the point of view of delivering the maximum amount of information to the following processing stages, therefore the features selected are considered *optimal*. The authors proposed that these optimal carriers of information are considered *salient* in fast vision and only these are used to build a saliency map^25^. Features that do not fulfill the constrained maximum-entropy optimization criterion are considered *non-optimal* and are not used to build the saliency map. Therefore, through the extraction of these *optimal* features, the system can compress information with minimal costs and provide a saliency map of the visual scene.

Here we adopt the same operational definition of saliency of a visual feature, that is determined by the amount of information^26^ it carries about the visual scene, weighed by the system processing costs.

In the original paper^13^, the authors implemented the model by using very small features to target early vision structures, known to have small receptive fields, and applied the model to black and white images^27^, for computational economy reasons. The model extracted a set of fine-scale, black and white *optimal* features, which closely resemble the structure (edges, lines etc.) of receptive fields found in primary visual cortices^28^, suggesting that they represent the result of such a constrained optimization of information-transmission process. On the other end, the features considered *non-optimal,* therefore discarded*,* consisted of “noisy” alternation of black and white pixels (features with large storage occupation) or had uniform luminance (all black/all white; features with large bandwidth occupation). Filtering natural images with the *optimal* features provided highly compressed *sketches* of visual scenes which resulted in accurate saliency maps of the originals^13^. *Optimal* features turned out to be arranged along objects’ contours (edges and lines) rather than being scattered throughout the image. Human participants’ performances in discriminating original images based on very brief presentations (25 ms) of these sketches was very accurate, comparable to that obtained by using their grey-scale original versions^13^. On the other end, filtering natural images with *non-optimal* features led to *sketches* that produced inaccurate discrimination.

Very recently, it has been shown that these *optimal* features are considered significantly more salient than others even if presented in isolation (not arranged along contours) for few milliseconds, without any clues coming from a global structure^29^. Participants, when explicitly asked to choose (with psychophysical and eye-movement tasks) the most salient stimulus, preferred *optimal* features, even when their number or contrast were lower than *non-optimal* features^29^.

In the present work, we aim to study the bottom-up saliency driven by these *optimal* features without explicitly requiring the participants to pay attention to stimulus saliency. We implicitly tested the relative saliency of *optimal* and *non-optimal* features by engaging participants in carrying out covert attentional and gaze-orienting tasks, whose performance might be influenced by the saliency of the task-irrelevant presented features.

Although the vast majority of spatially-cued attention orienting tasks (Posner paradigm^30^) have used a single cue (for a review see^5^), in our work, we designed a novel spatial-cueing task, in which two brief peripheral bilateral cues are presented before the target. Few studies have previously proven the efficacy of dual cues of different saliency (in terms of luminance contrast) in the automatic capture of attention toward the most salient one^31,32^. Here we use one *optimal* feature (deemed salient cue) and one non-*optimal* feature (deemed non-salient cue), which may preferentially attract the observer’s attention and eye movements to one location instead of another. Contrast-based saliency of cues was tested as a control for the saliency determined by the specific spatial structure of *optimal* features. That is, in the control condition, the saliency of the cues was manipulated through their relative contrast, presenting one high-luminance (deemed salient) and one low-luminance feature (deemed non-salient) as attentional cues^31^.

We measured covert attention and gaze-orienting performance with two different tasks in two experiments. The covert-attention task required to identify the orientation of a gabor presented with different contrasts in positions that were cued by an *optimal* or *non-optimal* feature. Exploiting the fact that attention automatically shifts to salient stimuli^33–35^, and that contrast sensitivity of stimuli presented in attended locations improves^36^, if *optimal* features are actually salient and able to automatically capture our attention, we expect lower contrast thresholds for targets presented in the position cued by one of them (“saliency cueing effect” in valid trials^5,30^). On the contrary, if the participants’ covert attention is captured on the opposite side of the target (invalid trials), the contrast threshold for the target discrimination should increase.

The gaze-orienting task only required making a saccade toward a visual target. Given that attentional capture to a specific location precede^37,38^ and facilitate a subsequent saccadic gaze shift to that location^38–40^, if *optimal* features are actually salient and capture our attention, we expect that saccades latencies will decrease towards targets presented in the position cued by one of them (valid trials). On the contrary, if the participants’ attention is captured on the opposite side of the target (invalid trials), the target-directed saccadic latencies will increase. In addition, since salient stimuli can elicit automatic short-latency saccades^41^, we might observe an automatic fast attraction of gaze (overt attention) exerted by our salient cues, irrespective to the target shown.

In both experiments, cueing features are presented for few milliseconds to probe early visual stages, implicated by the reference model.

In both tasks, the cue validity (i.e., the percentage of cases in which the target is presented in the position cued by the salient feature) could be 80% or 50%. The comparison between these two validity conditions, may help disentangling the nature of the attentional processes at play, since a facilitation effect based purely on exogenous attention, hence guided only by the stimulus properties and not willfully monitored^42^, should not increase with cue validity^43–45^. On the other hand, if some cognitive strategic process is at play, we might expect an increased facilitation in valid trials when cue validity is 80%, compared to when the salient cue is uninformative about the target position (cue validity 50%, for a review see^5^).

## Methods

### Covert-attention experiment

#### Participants

Sixteen young naïve adults (10 women, mean age = 27.8 ± 2.3 years) took part in the experiment. Eye movements were monitored on five of them in a separate control session. Written informed consent was obtained from all participants. The local ethics committee (*Comitato Etico Pediatrico Regionale—Azienda Ospedaliero-Universitaria Meyer—Firenze FI*) approved the experimental paradigm, which complied with the Declaration of Helsinki.

#### Experimental setup

Participants were tested individually in a dark room. Stimuli were presented through an ACER computer (Windows 7) using the Psychophysics Toolbox extensions for Matlab^46^. The experiment was displayed on a 120-Hz gamma-corrected CRT Silicon Graphics monitor (1024 × 768 pixel), subtending 38.5° × 29.5° of visual angle at a viewing distance of 57 cm. To control correct fixation, the left eye position was recorded with an EyeLink 1000 system (SR research—500 Hz).

#### Stimuli and conditions

In both experimental and control conditions, stimuli preceding the target consisted of two small cues (arrays of 9 × 9 pixels, 0.3 degrees), which could be equally salient (neutral trials) or one more salient than the other (non-neutral trials).

**Figure 1:**
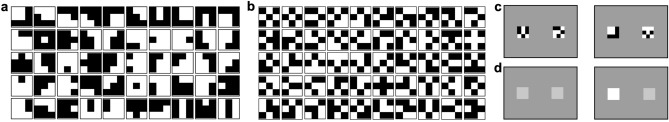
Stimuli and conditions. **(a)** *Optimal* features. Set of stimuli used as cues in the experimental condition of the attention tasks, considered as salient features by the reference model^13^. **(b)** *Non-optimal* features. Set of stimuli used as cues in the experimental condition of the attention tasks, considered non-salient features by the reference model^13^. On average, *optimal* and *non-optimal* features did not differ in luminance and in their spatial frequency content, between 9 and 27 cycles/deg, well above the frequency of maximum sensitivity (about 7 cycles/deg) in our illumination conditions^47^. **(c)** Experimental condition. Left panel:—Example of neutral trial. Two *non-optimal* features, considered equally non-salient by the reference model, used as baseline for the experimental condition. Right panel—Example of non-neutral trial. One *optimal* (left) and one *non-optimal* (right) feature, one more salient than the other according to the reference model. **(d)** Control condition. Left panel:—Neutral trial. Two cues with the same luminance contrast, considered equally salient, used as baseline for the control condition. Right panel—Example of non-neutral trial. Two cues with different luminance contrasts, one being considered more salient than the other based on their luminance. Features are shown oversized for illustration purposes. The cue stimuli and conditions used in the covert attention and gaze-orienting tasks were the same.

In the experimental condition, cues saliency was based on the spatial arrangement of their black (luminance 4 cd/m^2^) and white (44 cd/m^2^) pixels. Two types of cues were used: *optimal* features and *non-optimal* features*,* as identified by the reference constrained maximum-entropy model^13^. At each trial, the *optimal* feature, used as “salient cue”, was randomly extracted from a set of 50 features selected as the best information-carriers (Fig. 1a); whereas the *non-optimal* feature, used as “non-salient cue”, was extracted from a set of 50 features selected amongst those with the lowest probability of occurrence in the statistical distribution of all possible features (Fig. 1b). Neutral trials (Fig. 1c—Left Panel) consisted of the presentation of two *non-optimal* features, deemed as two equally non-salient cues. In non-neutral trials (Fig. 1c—Right Panel), one *optimal* and one *non-optimal* feature were presented, that is one cue deemed as more salient than the other according to the reference model. A comparison between the saliency of these two types of features was assessed in past experiments^29^ where subjects preferred one optimal feature to a non-optimal in 70% of cases and ten *optimal* features were still preferred when their luminance-contrast was only 65% than that of ten *non-optimal* features.

In the control condition, the saliency of the cues was exploited through their relative luminance contrast. Neutral trials (Fig. 1d—Left Panel) consisted of the presentation of two identical grey features with equal luminance (20 cd/m^2^), slightly higher than that of the background, and therefore equally non-salient cues. In non-neutral trials (Fig. 1d—Right Panel), one high-luminance and one low-luminance feature were presented, that is one salient and one non-salient cue based on their different luminance. To compare the effect of different cue types on equal grounds, in the control condition the luminance values were set to 20 cd/m^2^ and 23 cd/m^2^. These values produce a contrast corresponding to the “equivalent contrast” found to match the saliency difference between *optimal* and *non-optimal* features^29^.

#### Procedure

**Figure 2:**
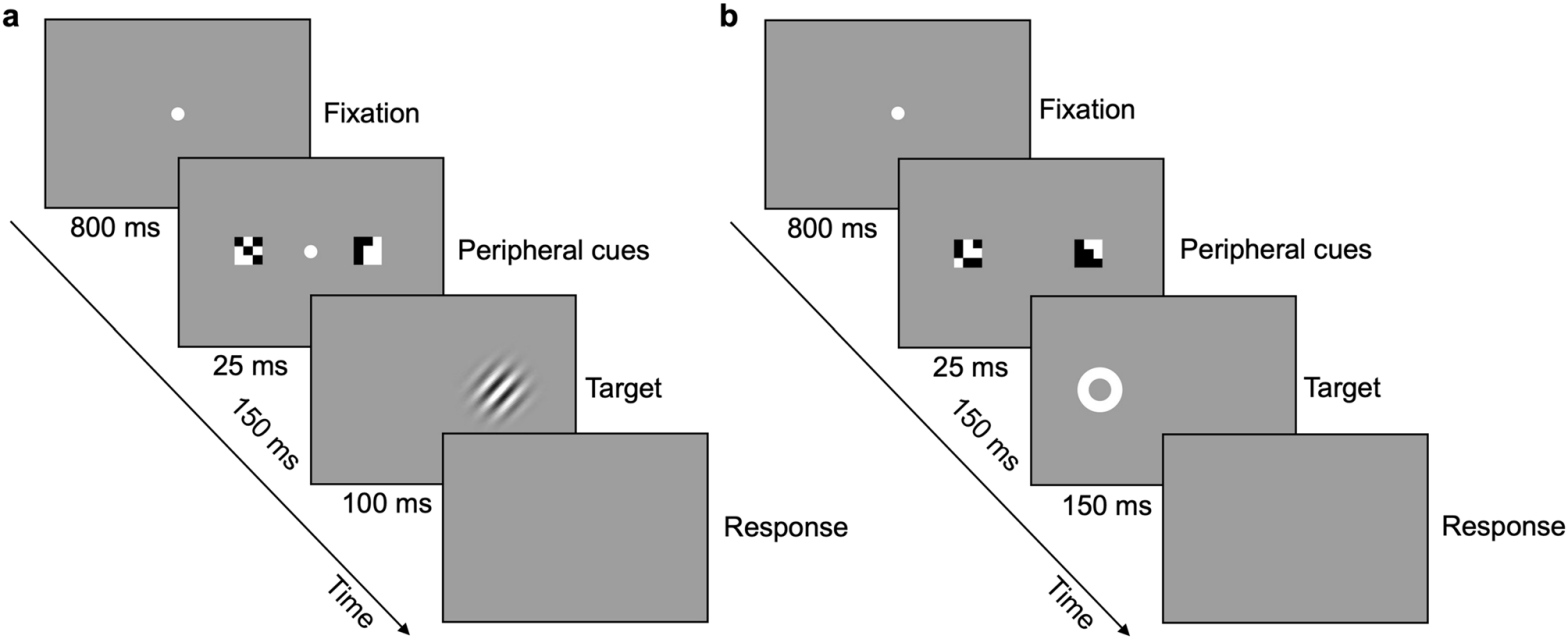
Procedure. **(a)** Covert-attention task. Example of non-neutral trial of the experimental condition, in which the *optimal* feature (the one on the right) precedes the target presentation (valid trial). Target is a ± 20°-tilted gabor (40 pixels large, 1.5 cm diameter, spatial frequency of 2 cycles per degree, sigma of 5.7, phase of 0.5π). **(b)** Gaze-orienting task. Example of non-neutral trial of the experimental condition, in which the *optimal* feature (the one on the right) is presented on the opposite side of the saccadic target (invalid trial). Target is a circular white placeholder, 100% contrast, 9 pixels large (0.3 cm diameter). Cues and targets are shown oversized for illustration purposes.

Each trial (Fig. 2a) started with the presentation of a grey display (16 cd/m^2^) with a central fixation point, followed by two peripheral cues, bilaterally presented at 5° of eccentricity. After 150 ms of SOA, a tilted gabor appeared at the same location of one of the two cues. The task instructions, given to the participants at the beginning of the experimental session by the experimenter, required them to discriminate the orientation of the gabor (i.e., clockwise or anticlockwise, communicated by a button press) while maintaining fixation. Eight different gabor contrasts, in the range between 0.01 and 0.09, were tested, presented in random order according to a constant stimuli procedure. The contrast values were slightly different across observers based on preliminary rough estimates of individual thresholds. Reaction times were also measured.

In both experimental and control conditions, 224 neutral and 500 non-neutral trials were presented (for examples see Fig. 1c,d). In neutral trials, the two cues were equally salient and uninformative about the position of the following target. Non-neutral trials could be “valid trials” or “invalid trials”, based on the position of the (deemed) salient cue with respect to the following target. In valid trials, the salient cue (*optimal* feature or high-luminance cue) was presented at the same location of the target, whereas, in the invalid trials, the salient cue was presented on the opposite side of the gabor, which therefore appeared after the non-salient cue (*non-optimal* feature or low-luminance cue).

The percentage of cases in which the target was presented in the position cued by the salient feature was defined as “cue validity”. 250 non-neutral trials have 50% cue validity (125 valid trials and 125 invalid) and the other 250 trials have 80% cue validity (200 valid trials and 50 invalid).

Data for each participant were collected in two sessions, one for each cue validity, performed in random order across participants. In each session, participants performed one block of neutral trials and two blocks of non-neutral trials, one for the experimental and one for the control condition, presented in random order. Each participant performed 1448 trials in total.

A subset of participants performed an additional separate session with one block of neutral trials and one block of non-neutral trials with 80% cue validity while their eye movements were recorded. This session allowed us to control that the results obtained in the main covert-attention task were not due to uncontrolled saccades toward the salient cue, which could potentially reduce the perceptual threshold by reducing the distance of the gabor from the fovea.

#### Data processing

Percent correct data were fitted (MLE) with a cumulative Gaussian error function. For each participant, condition (experimental and control), cue validity (50% and 80%), and trial type (neutral, valid, and invalid) thresholds were calculated as the target contrasts yielding 80% correct responses. Thresholds for neutral trials of each condition were used as baseline for non-neutral trials. That is, they were subtracted from those obtained in valid and invalid trials, and the result divided by them to provide a measure of the percentage increase or decrease of contrast thresholds in non-neutral trials.

In the control session, we considered a saccade execution any shift of gaze position further than 2 degrees from the fixation point.

### Gaze-orienting experiment

#### Participants

Sixteen naïve young adults (11 women, mean age = 27.6 ± 1.9 years) participated in the experiment. Written informed consent was obtained from all participants (all different from those of the covert-attention experiment). The local ethics committee (*Comité d’éthique d’Aix-Marseille Université, ref: 2014-12-3-05*) approved the experimental paradigm, which complied with the Declaration of Helsinki.

#### Experimental setup

Each participant was tested individually in a dark room. Stimuli were presented through a MacPro computer (OS 10.6.8), using the Psychophysics Toolbox^46^ and the Eyelink Toolbox extensions^48^ for Matlab. The experiment was displayed on a 120-Hz CRS Display++ LCD monitor (1920 × 1080 pixel), subtending 70 × 40 degrees at a viewing distance of 57 cm. Participants’ viewing was binocular, but only the right eye was recorded by an Eyelink 1000 video-based eye tracker (1 kHz). The observers’ head was stabilized with a chin- and forehead rest.

#### Stimuli and conditions

Cue stimuli and conditions were the same as in the covert-attention experiment.

#### Procedure

Each trial (Fig. 2b) started with the presentation of a grey display (16 cd/m^2^), with a central fixation point, followed by two peripheral cues, bilaterally presented at 5° of eccentricity. After 150 ms of SOA, the target appeared on the left or on the right of the center, in the same location of one of the two cues. At the beginning of the experimental session, participants were instructed to make a saccade towards the target as quickly as possible. The following trial started only after participants resumed fixation.

In both experimental and control conditions, 200 neutral and 500 non-neutral trials were presented. 250 non-neutral trials have 50% cue validity, the other 250 trials have 80% cue validity.

Data were collected in two sessions, one for each cue validity. In each session, 100 neutral trials and 250 non-neutral trials for each condition were tested. Each participant performed 1400 trials in total.

#### Data processing

Oculomotor parameters were extracted by using ad hoc software in Matlab. Recorded horizontal and vertical gaze positions were low-pass filtered with a Butterworth (acausal) filter of order 2 with a 30-Hz cutoff frequency and then numerically differentiated to obtain velocity measurements. An automatic conjoint acceleration and velocity threshold method was used to detect saccades^49^. Aberrant trials, without recorded saccades (e.g., due to a long blink), were excluded (less than 3% of all saccades).

In each trial, we considered a “*regular saccade*” the first detected saccade with a latency (with respect to target onset) longer than 80 ms^50,51^ and shorter than 500 ms (~ 95% of the first detected saccades overall), and an amplitude larger than 2 degrees (40% of the entire eccentricity). For each regular saccade, we estimated latency and direction. Each regular saccade was labeled as “*correct*” if directed to the target or as “*erroneous*” if directed towards the opposite side of the target. The mean latencies of *correct* saccades calculated in neutral trials of each condition were used as baseline for non-neutral trials. That is, they were subtracted by the latency values obtained in valid and invalid trials, and the result divided by them, yielding to a measure of the percentage increase or decrease of saccadic latencies. The percentage of saccade direction errors relative to the total number of saccades in each condition was also measured.

Trials’ inspection revealed a consistent number of saccades faster than 80 ms. These values are considered too fast to be due to the onset of the target^50^ and are probably generated by the presentation of the cues. Therefore, saccades with latency shorter than 80 ms and longer than − 70 ms (with respect to the target onset, corresponding to a latency of 80 ms with respect to cue onset), and amplitude of at least 1 degree (a widely used arbitrary threshold-amplitude to exclude fixational microsaccades^52^), were categorized as “anticipatory saccades”, potentially elicited by the cue. In most cases, an anticipatory saccade preceded a regular saccade, which continued in the same direction or reversed direction to reach the target. The percentage of anticipatory saccades over the total number of saccades in each condition was measured. Since not all participants performed anticipatory saccades, weighted averages were computed, taking into account their number in each condition, and percentages over the number of anticipatory saccades in non-neutral trials were calculated to estimate their preferential direction.

## Results

### Covert-attention experiment

#### Contrast thresholds

**Figure 3:**
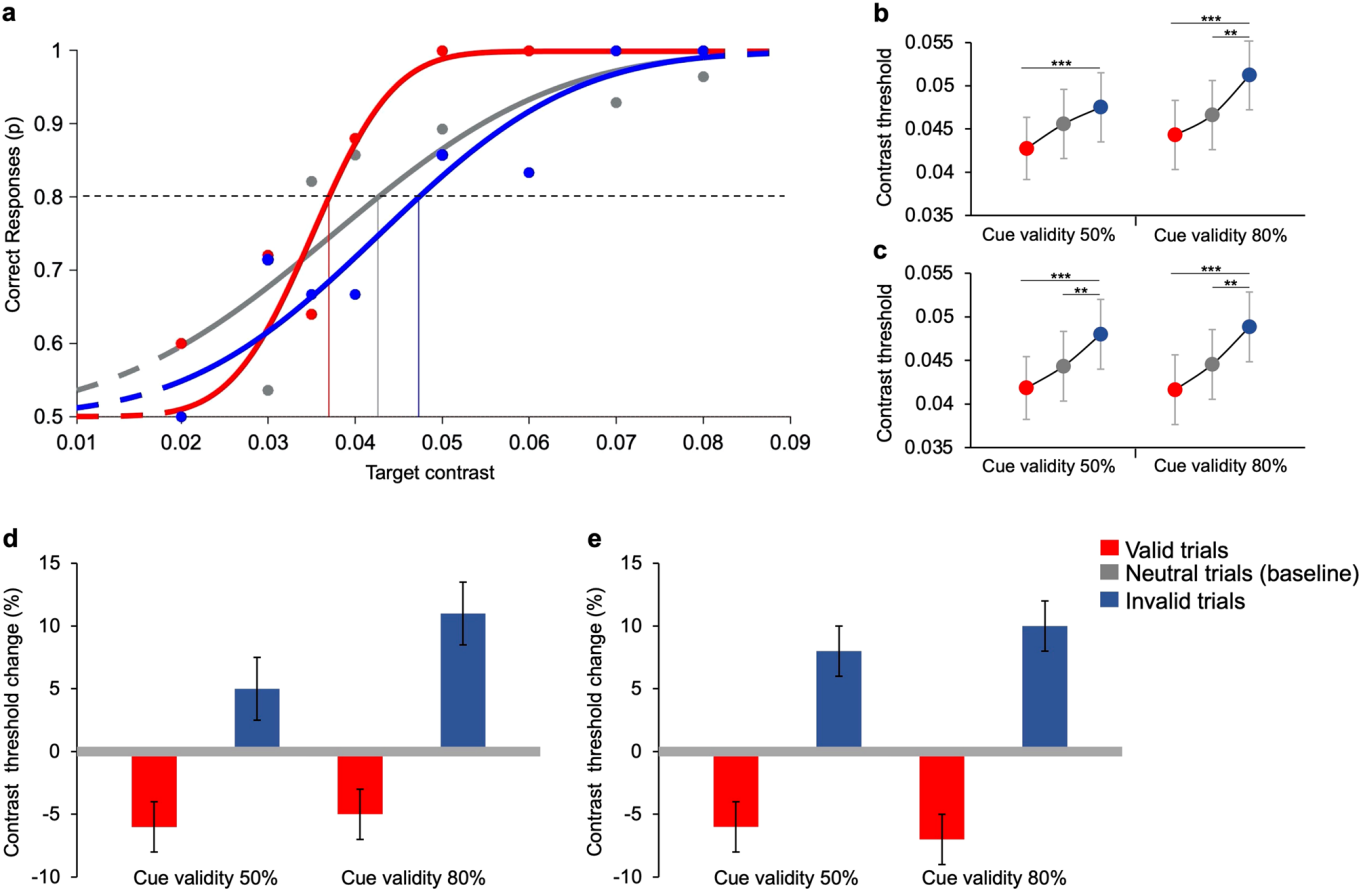
Contrast thresholds in valid, invalid, and neutral trials. **(a)** Example of correct responses as a function of target contrast for one participant. Performance obtained in the experimental condition with 80% cue validity. Data are obtained from 224 neutral trials, 200 valid trials, and 50 invalid trials. Filled circles represent the proportion of times in which the participant correctly discriminated the gabor orientation presented with a specific contrast. The curves represent cumulative Gaussian error fits of the data. The vertical lines represent contrast thresholds, i.e., contrast values yielding 80% correct responses (dashed line). The color coding of dots, curves and lines is red for valid, blue for invalid, and grey for neutral trials. **(b)** Experimental condition. Group average contrast thresholds in neutral (grey), valid (red), and invalid (blue) trials. Post-hoc *t*-tests (Bonferroni correction) show a significant difference between valid versus invalid trials for 50% cue validity (*t*(2) = 5.12, *p* < 0.001), They show also significant differences between valid versus invalid trials (*t*(2) = 6.05, *p* < 0.001), and invalid versus neutral trials (*t*(2) = − 4.07, *p* = 0.002) for 80% cue validity. **(c)** Control condition. Group average contrast thresholds in neutral (grey), valid (red), and invalid (blue) trials. Post-hoc t-tests (Bonferroni correction) show a significant difference between valid versus invalid trials (*t*(2) = 6.52, *p* < 0.001), and invalid versus neutral trials (*t*(2) =  − 3.73, *p* = 0.006) for 50% cue validity. They also show significant differences between valid versus invalid trials (*t*(2) = 6.26, *p* < 0.001), and invalid versus neutral trials (*t*(2) =  − 3.64, *p* = 0.009) for 80% cue validity. Asterisks mark statistically significant pairwise comparisons across trial types: **p < 0.01, ***p < 0.001. Error bars are SEM. **(d)** Experimental condition. Group average threshold changes in valid (red) and invalid (blue) trials compared to the baseline (grey line, neutral trials with two *non-optimal* features). **(e)** Control condition. Group average threshold changes in valid and invalid trials compared to the baseline (neutral trials with two identical low-luminance grey features).

The performance of one participant for valid, invalid, and neutral trials in the experimental condition is shown in Fig. 3a, as an example. At the lowest contrast performance is at chance level and then increases with target contrast in all trial types. However, performance is higher in valid trials than in neutral trials. Invalid trials have the lowest performance. This result holds true for all our participants.

Contrast thresholds averaged over 16 participants are reported in Fig. 3b **(**experimental condition),c (control condition). In the experimental condition, average gabor’ contrast thresholds for blocks with 50% cue validity are 0.045 ± 0.004 (SEM), 0.042 ± 0.004, and 0.048 ± 0.004 for neutral, valid, and invalid trials, respectively. For blocks with 80% cue validity average thresholds are 0.047 ± 0.004, 0.044 ± 0.004, and 0.051 ± 0.004 for neutral, valid, and invalid trials, respectively. Average percentage threshold changes in valid and invalid trials compared to baseline values for the experimental condition are reported in Fig. 3d. Contrast thresholds with respect to baseline decrease in valid trials and increase in invalid trials, both for 50% (− 6.19 ± 2% and + 5.24 ± 2.5%, respectively) and 80% cue validity (− 4.81 ± 2.2% and + 10.72 ± 2.8%, respectively).

Results for the luminance control condition are very similar: averaged target contrast thresholds for 50% cue validity are 0.044 ± 0.004, 0.041 ± 0.005, and 0.048 ± 0.005 in neutral, valid and invalid trials, respectively. Average contrast thresholds for 80% cue validity are 0.045 ± 0.004, 0.042 ± 0.004 and 0.049 ± 0.004 in neutral, valid and invalid trials, respectively. Average relative threshold changes in the control condition are reported in Fig. 3e. As in the experimental condition, percentage contrast thresholds with respect to baseline decrease in valid trials and increase in invalid trials, both for 50% (− 6.41 ± 2.3% and + 8.69 ± 2.1%, respectively) and 80% cue validity (− 6.80 ± 2.4% and + 10.39 ± 2.2%, respectively).

Although the differences between means are small, three-ways ANOVA analysis—with factors: condition (two levels: experimental vs. control), trial type (three levels: neutral, vs. valid, vs. invalid), and cue validity (two levels: 50% vs. 80%)—evidences a significant main effect of type of trial (F(2, 30) = 67.53, *p* < 0.001, η^2^ = 0.03 – *small* effect size) but no effect of either condition (F(1,15) = 1.03, *p* = 0.3, η^2^ = 0.002) or cue validity (F(1,15) = 1.84, *p* = 0.19, η^2^ = 0.001) on the perceptual threshold. No interactions between the three factors have been found. Pairwise comparisons *t*-tests (with Bonferroni corrections), performed to assess significant differences between the means of different trial types are reported in the caption of Fig. 3.

Average contrast thresholds changes of participants in the 50% cue validity condition were not statistically different depending on whether this condition was performed before or after the 80% cue validity condition (Independent *t*-test—experimental condition: valid: *t*(14) =  − 0.32, *p* = 0.7, invalid: *t*(14) = 0.47, *p* = 0.6; control condition: valid: *t*(14) =  − 0.67, *p* = 0.5, invalid: *t*(14) =  − 0.60, *p* = 0.6), thus arguing against a prominent role for sessions’ order.

Reaction times analysis showed no differences between valid and invalid trials; indeed, in all types of trials, conditions and cue validities, average reaction times are very long (~ 600 ms).

In the additional session of the experimental condition with 80% cue validity, in which observers’ fixation was monitored, contrast thresholds change was − 5.46 ± 2.7% in valid trials and + 10.7 ± 2.7% in invalid trials, comparable to those obtained in the first participation without fixation control (respectively: − 5.6 ± 2.8%, + 13.4 ± 4.6%). On average, only one or two saccades over 474 trials were detected in these observers.

### Gaze-orienting experiment

#### Saccadic latencies

**Figure 4:**
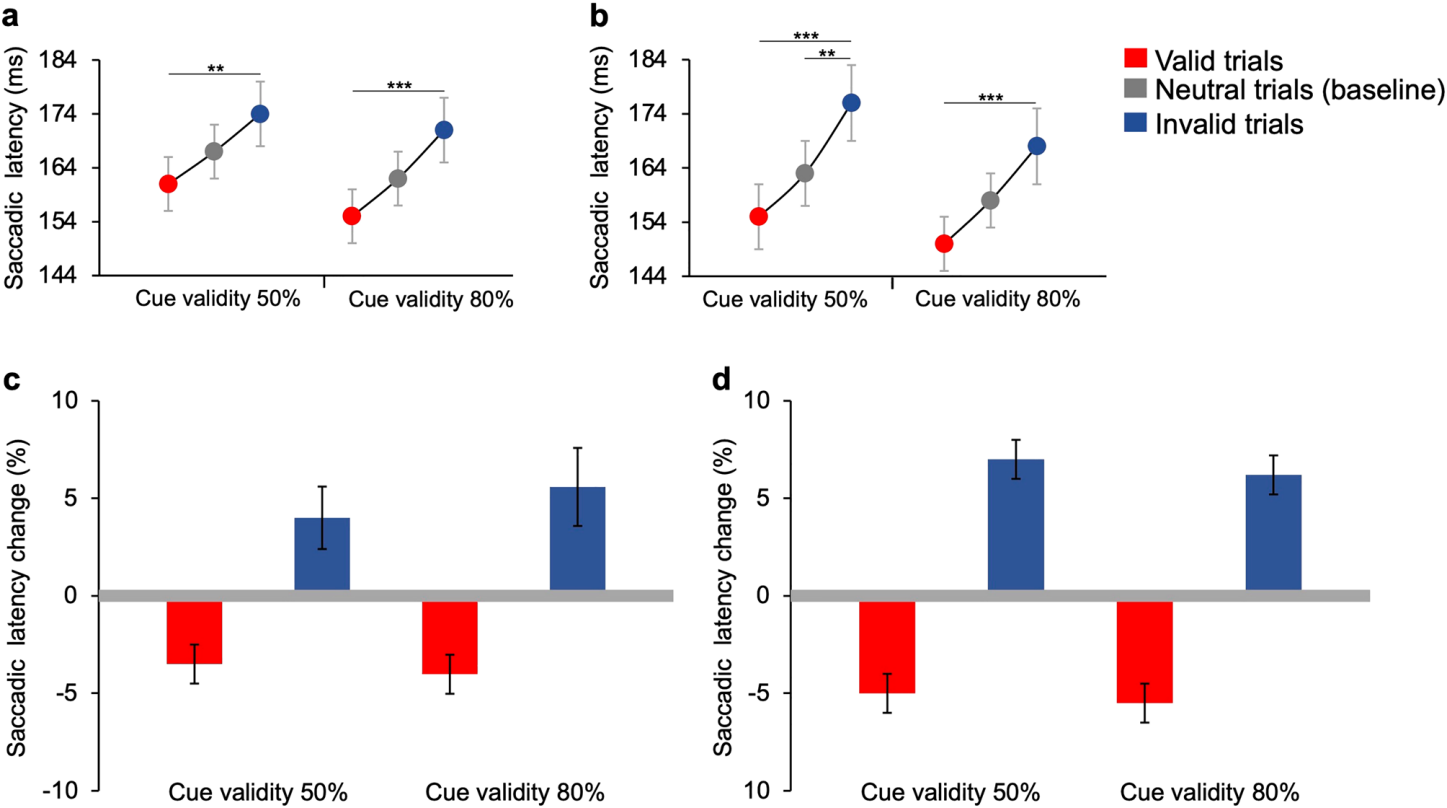
Saccadic latencies in neutral, valid, and invalid trials. **(a)** Experimental condition. Group average latencies in neutral (grey), valid (red), and invalid (blue) trials. Post-hoc t-*t*ests (Bonferroni correction) show a significant difference between valid versus invalid trials for 50% (*t*(2) = 3.99, *p* = 0.008) and 80% cue validity (*t*(2) = 5.21, *p* < 0.001). **(b)** Control condition. Group average latencies in neutral (grey), valid (red), and invalid (blue) trials. Post-hoc *t*-tests (Bonferroni correction) show significant differences between valid versus invalid trials (*t*(2) = 6.72, *p* < 0.001), and invalid versus neutral trials (shown with line near the baseline; *t*(2) =  − 4.05, *p* = 0.007) for 50% cue validity. They also show a significant difference between valid versus invalid trials (*t*(2) = 6.16, *p* < 0.001) for 80% cue validity. Asterisks mark statistically significant pairwise comparisons across trial types: **p < 0.01, ***p < 0.001. Error bars are SEM. **(c)** Experimental condition. Group average latencies changes in valid (red) and invalid (blue) trials compared to the baseline (grey line, neutral trials with two *non-optimal* features). **(d)** Control condition. Group average latencies changes in valid and invalid trials compared to the baseline (neutral trials with two identical low-luminance grey features).

Latencies of *regular* saccades directed to the target (*correct*) differ across different types of trials. Saccadic latencies averaged over 16 participants are reported in Fig. 4a (experimental condition),b (control condition).

In the experimental condition, for 50% cue validity, average latencies are 167 ± 5.9 ms (SEM), 161 ± 5.7 ms, and 174 ± 6.7 ms in neutral, valid and invalid trials, respectively. Average latencies for 80% cue validity are 162 ± 5.3 ms, 155 ± 5.6 ms, and 171 ± 6.1 ms in neutral, valid and invalid trials, respectively. The average latency changes in valid and invalid trials relative to baseline values are reported in Fig. 4c. Percentage latencies changes relative to baseline decrease in valid trials and increase in invalid trials, both for 50% (− 3.5 ± 1% and + 3.7 ± 2%, respectively) and 80% cue validity (− 4.1 ± 1% and + 5.5 ± 2%, relatively).

Results of the luminance control condition are comparable to those of the experimental condition. The mean saccadic latency is 163 ± 6.6 ms in neutral trials, 155 ± 6.4 ms in valid trials, and 176 ± 7.5 ms in invalid trials, for blocks with 50% cue validity. For blocks with 80% cue validity mean saccadic latency is 158 ± 5.5 ms in neutral trials, 150 ± 5.4 ms in valid trials, and 168 ± 7.2 ms in invalid trials. Average percentage latency changes are reported in Fig. 4d**.** Percentage latencies with respect to baseline decrease in valid trials and increase in invalid trials (50% cue validity: − 4.9 ± 1% and + 7.4 ± 1%, respectively; 80% cue validity: − 5.5 ± 1% and + 6.20 ± 1%, respectively). Also in this case, results for blocks with 50% cue validity are similar to those obtained for 80% cue validity.

Three-ways ANOVA analysis—with factors: condition (two levels: experimental vs. control), trial types (three levels: neutral, vs. valid, vs. invalid), and cue validity (two levels: 50% vs. 80%)—evidences a significant main effect of types of trial (F(2,30) = 34.11, *p* < 0.001, η^2^ = 0.08—*small* effect size) but no effect of either condition (F(1,15) = 2.3, *p* = 0.15, η^2^ = 0.005) or cue validity (F(1,15) = 2.69, *p* = 0.12, η^2^ = 0.01) on saccadic latency. No interactions between the three factors have been found. Pairwise comparisons *t*-tests (with Bonferroni corrections), performed to assess significant differences between the means of the different trial types, are reported in the caption of Fig. 4.

A possible effect of sessions’ order does not seem very likely. Indeed, averaged saccadic latency changes in the 50% cue validity condition did not change significantly if the participants had first performed the session with 80% cue validity or the other way around (Independent *t*-test—experimental condition: valid: *t*(14) = 1.07, *p* = 0.3, invalid: *t*(14) = 0.76, *p* = 0.4; control condition: valid: *t*(14) = 0.50, *p* = 0.6, invalid: *t*(14) = 0.37, *p* = 0.7).

### Saccadic direction errors

**Figure 5:**
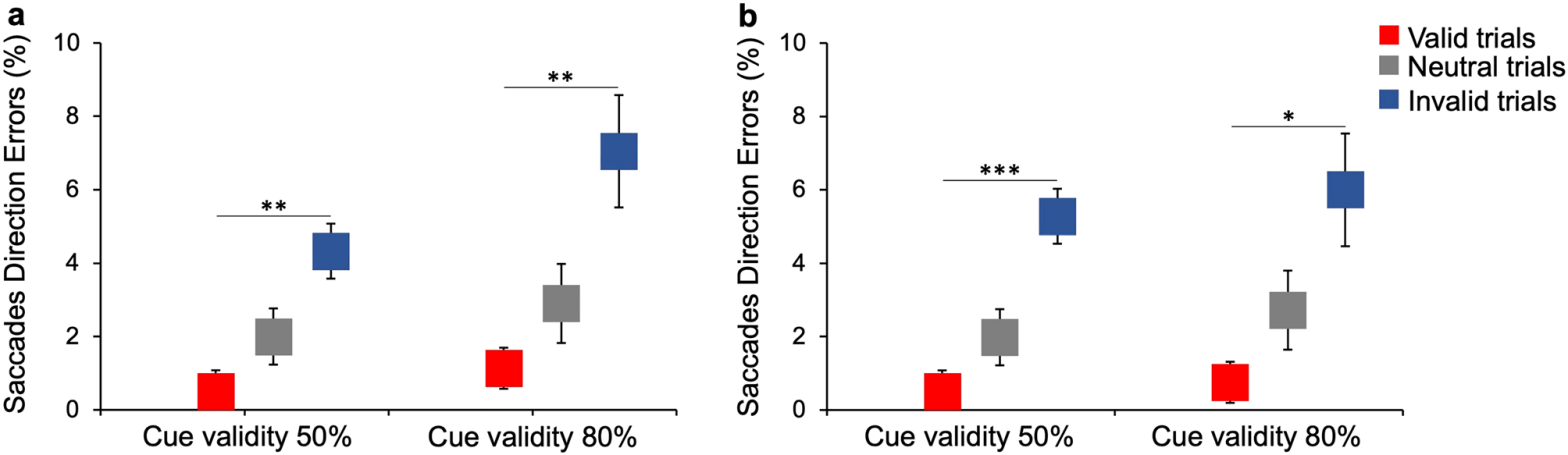
Percentage of saccadic direction errors in neutral, valid, and invalid trials. **(a)** Experimental condition. Post-hoc Conover tests (Bonferroni corrections) show that the percentage of saccades direction errors in valid trials (red) is lower than that in invalid trials (blue) for 50% (*t*(11) = 4.05, *p* < 0.01) and 80% cue validity (*t*(11) = 4.02, *p* < 0.01). **(b)** Control condition. Post-hoc Conover tests (Bonferroni correction) show that the percentage of saccades direction errors in valid trials is lower than that in invalid trials for 50% (*t*(11) = 5.02, *p* < 0.001) and 80% cue validity (*t*(11) = 3.56, *p* < 0.05). Asterisks mark statistically significant pairwise comparisons across trial types: *p < 0.05; **p < 0.01; ***p < 0.001. Error bars are SEM.

In the gaze-orienting task, although participants were instructed to make an accurate saccade towards the visual target, there was a small proportion of trials in which the participants moved their eyes towards the opposite side of the target (*erroneous* saccades). Independently on the condition and the cue validity, a small percentage of direction errors relative to the total number of saccades is present in all trial types, even in neutral trials, in which the cues preceding the target were equally salient. Interestingly, the proportion of erroneous saccades decreases in valid trials and increases in the invalid ones with respect to neutrals (baseline) (Fig. 5). Specifically, in the experimental condition with 50% cue validity (Fig. 5a, left), there are, on average, 2.2 ± 0.8% (SEM) erroneous saccades in neutral trials, and only 0.4 ± 0.1% in valid trials. Instead, in invalid trials, there are 4.3 ± 0.7% direction errors. Similarly, in trials with 80% cue validity (Fig. 5a, right), there are 2.9 ± 1.1%, 1.1 ± 0.5%, and 7.1 ± 0.5% errors in neutral, valid and invalid trials, respectively.

The same pattern of results holds for the control condition. In trials with 50% cue validity (Fig. 5b, left), there are 2 ± 0.9%, 0.2 ± 0.1% and 5.3 ± 1% direction errors in neutral, valid and invalid trials, respectively. In trials with 80% cue validity **(**Fig. 5b, right), there are 2.7 ± 0.8%, 0.8 ± 0.3%, and 6 ± 1.3%, direction errors in neutral, valid and invalid trials, respectively.

Friedman non-parametric test (for binomial distributed data) confirms that there is an effect of trial type and that the proportion of direction errors is significantly higher in invalid trials compared to valid ones (χ2(11) = 78.08, *p* < 0.001, W = 0.3). Pairwise comparisons with Conover post-hoc tests (with Bonferroni corrections), performed to assess significant differences between the means of the different trial types, are reported in the caption of Fig. 5.

### Anticipatory saccades

**Figure 6:**
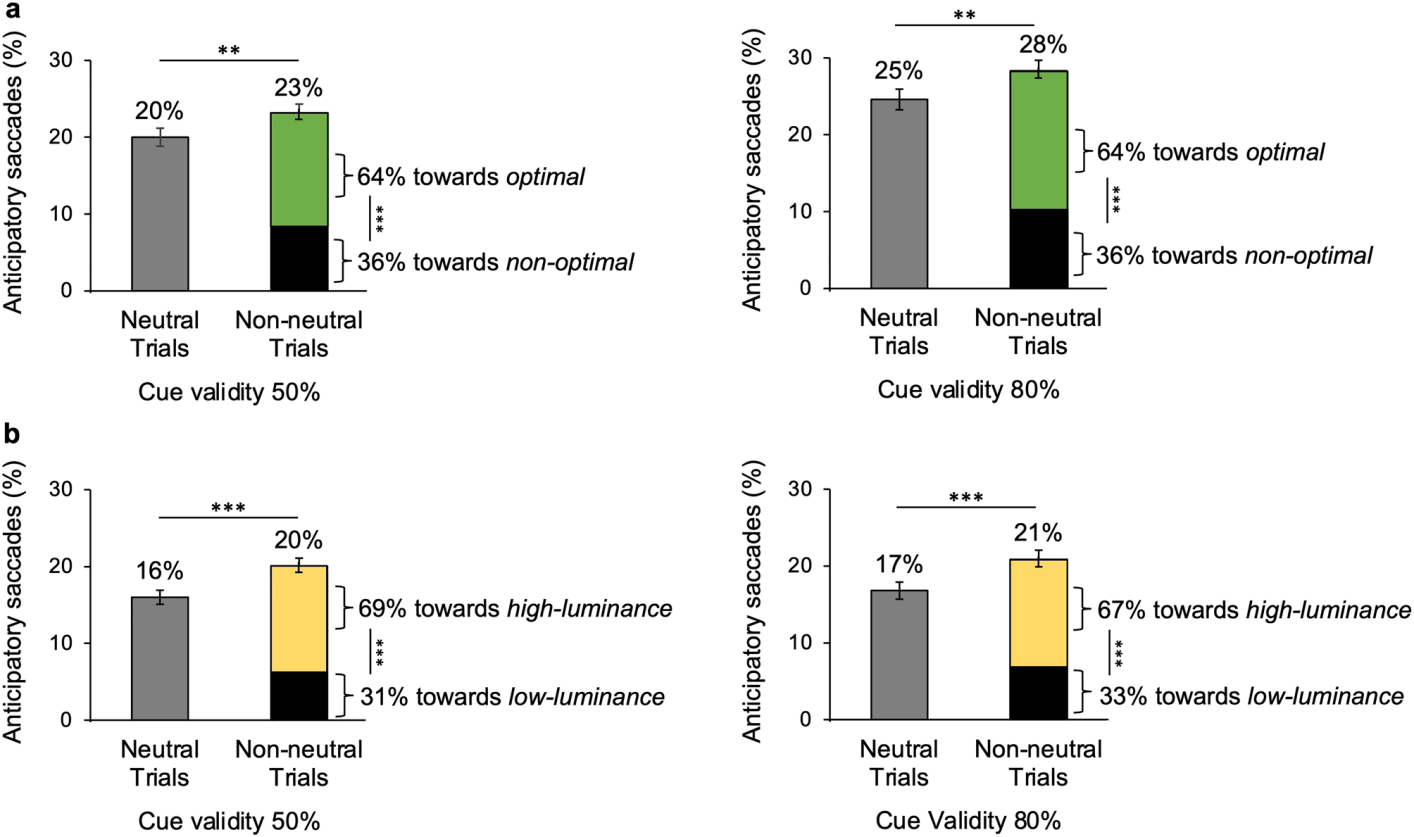
Percentage and direction of anticipatory saccades in neutral and non-neutral trials. Percentages shown above bars are computed over the total number of saccades. Percentages shown on the sides of green/black and yellow/black bars are computed over the number of anticipatory saccades done in non-neutral trials and represent the preferential direction towards which these saccades are directed. Binomial data were considered as normally distributed due to the numerosity of observations for each trial type (> 30). **(a)** Experimental condition. The percentage of anticipatory saccades in non-neutral trials (green/black bars) is higher than that in neutral (grey bars), for 50% (left panel; *z* = 2.33, *p* = 0.009) and 80% cue validity (right panel; *z* = 2.70, *p* = 0.003). Anticipatory saccades in non-neutral trials are preferentially directed to the *optimal* feature (green) compared to the *non-optimal* one (black) (50% cue validity—*z* = 11.27, *p* < 0.001; 80% cue validity—*z* = 12.57, *p* < 0.001). **(b)** Control condition. The percentage of anticipatory saccades in non-neutral trials (yellow-black bars) is higher than that in neutral (grey bars), for 50% (left panel; *z* = 3.42, *p* < 0.001) and 80% cue validity (right panel; *z* = 3.30, *p* < 0.001). Anticipatory saccades in non-neutral trials are preferentially directed to the high-luminance feature (yellow) compared to the low-luminance one (black) (50% cue validity: *z* = 14.39, *p* < 0.001; 80% cue validity: *z* = 13.22, *p* < 0.001). Asterisks mark statistically significant pairwise comparisons across trial types: **p < 0.01, ***p < 0.001. Error bars are SEM.

As shown in Fig. 6, in each condition (experimental and control) and cue validity (50% and 80%), there is a high percentage of anticipatory saccades with respect to the total number of saccades, that is very early saccades with respect to stimulus onset. Statistical analyses, reported in the caption of Fig. 6, show that the percentage of anticipatory saccades changes across trial types. Not surprisingly, the number of anticipatory saccades is always higher in non-neutral trials (i.e., valid and invalid trials) compared to neutral trials (percentages reported over each vertical bar in Fig. 6). No differences across experimental and control conditions emerge. In the experimental condition there are slightly more anticipatory saccades for 80% than 50% cue validity, whereas there are no differences across cue validities for the control condition.

Anticipatory saccades in non-neutral trials clearly show a preferential direction. In the experimental condition, they are mainly directed to the *optimal* feature, with no difference between cue validities. A similar percentage of preferential direction is obtained in non-neutral trials of the control condition, where anticipatory saccades are mainly directed to the high-luminance feature, again not depending on cue validity.

## Discussion

In the present study, we test the automatic capture exerted by a specific set of local features deemed salient, originally identified as optimal information carriers based on constrained-entropy maximization criteria^13^. Previous works have shown that *optimal* features improve discrimination, when embedded in simplified sketches of natural images^13^. Also, explicit preference for *optimal *versus* non-optimal* features presented in isolation, without any global arrangement, was recently demonstrated^29^. Here, the *optimal* features saliency is implicitly addressed by measuring participants’ performance in perceptual and oculomotor dual-cueing attentional tasks where they are used as cues.

Results of the covert-attention task show that when the target is cued by an *optimal* feature its contrast threshold decreases, while when the target is presented on the opposite side of the *optimal* cue its contrast threshold increases. Since contrast sensitivity improves at attended locations^36^, the effect found here could be attributed to the attentional capture of *optimal* features towards the location where they are shown, being more salient than the others. Since this effect is not due to eye movements to the target, it can be attributed to covert attention.

The saliency-based capture of *optimal* features is also seen in the overt response in the gaze-orienting task. First, latencies of *regular* saccades towards the target decrease when cued by *optimal* features. As the dynamics of ocular movements is known to be influenced by attentional factors^38,39^, this can be seen as indication that *optimal* features are perceived as a potentially salient stimulus to be analyzed. Saccades directed to the target cued by *non-optimal* feature are slower, probably because the system had already allocated attention and was ready to direct the gaze on the opposite side and has therefore to re-allocate its resources.

The attentional capture exerted by the *optimal* features is also reflected in the number of errors in saccade direction. We find that some *regular* saccades were not directed to the target, despite task requirements. When the target is cued by an *optimal* feature very few errors occur, compared to when the position of the *optimal* cue and the target do not match. This indicates that an *optimal* feature attracts the participant's attention overtly, triggering a saccade to a location, that in some trials does not allow a correction based on the target location, resulting in the gaze landing on the opposite side of the target.

Finally, anticipatory saccades, which are considered too fast to be due to the onset of the target and are probably generated by the presentation of the cues^50^, are more numerous when the two cues differ in saliency (non-neutral trials with an *optimal* and a *non-optimal* cue) rather than when they are equally salient (neutral trials, two *non-optimal* cues). This fast oculomotor response might be due to an imbalance of mutual inhibition between neural populations representing the two locations, possibly occurring in the superior colliculus^53^. Moreover, in non-neutral trials, anticipatory saccades are mainly directed to the side where the *optimal* feature is presented, providing further support for the fast, automatic attraction exerted by the *optimal* features.

Overall, the results of our experiments reveal the presence of a “saliency-based cueing effect”, in which the participants' covert and overt attention is attracted by the *optimal* features. That is, *optimal* features result to be used as salient attentional cues by our visual system.

In both tasks and conditions, there is no evidence of an increase of the attention-grabbing effect with cue validity. This is in agreement with most other studies, showing that, unlike endogenous attention, exogenous attention is automatic and unaffected by cue validity^5,43^; that is, attention capture by *optimal* features’ seems to be automatic and guided by the exogenous properties of the features. However, the peripherical cue position and the brevity of the SOA may have precluded an emergence of endogenous effects, which are usually manipulated by central cues and need more time to occur compared to exogenous effects^5,43,54,55^. Kean et al. (2003)^31^ have found a somewhat counterintuitive effect, whereby attention is captured by the most salient cue only when the cues are irrelevant to task (i.e., cue validity 50%), but not when they indicate the critical location for attentional allocation to the target (i.e., cue validity 80%). However, their participants were informed of the contingent relationship between the bright cue and the target location and expectancy has been shown to attenuate the automatic attention capture^56^. Our participants were completely unaware of the cues’ predictivity (or non-predictivity) relative to the goal location, so attention capture is not expected to decrease.

The vast majority of the studies investigating spatial-cueing effects on automatic capture of attention have used a single peripheral cue (for a review, see^5,57^). Here instead we chose to present two simultaneous peripheral cues, with either the same or different assumed saliency, to test the power of the model-predicted *optimal* features in an implicit competition to attract the participants’ attention. To our knowledge, only one other study has used this dual-cue paradigm to study gaze orienting task with luminance-based cues^31^. This dual cues saliency manipulation is also directly comparable with that used in a recent study of our group^29^, where participants could discriminate between the saliency of two stimuli, very similar to those used here, but, unlike here, were explicitly asked to do so. Given our result, such a double spatial-cueing paradigm may be a useful general tool to test the saliency of two stimuli, also ontologically different from each other, by directly comparing their ability to capture attention. Note however that, the latency advantage found here is similar to that elicited by a single uninformative cue versus a non-cued target location^58–60^, under comparable experimental conditions and at short SOAs. These findings seem to suggest that the more salient of two peripheral cues elicits an attention-capturing effect of a similar magnitude to that of a single peripheral stimulus^31^.

Very interestingly, in both tasks, all the effects found with *optimal *versus* non-optimal* features are comparable to those obtained with cues of different luminance. This suggests that the saliency provided by *optimal* features is comparable to that of high-luminance cues, if compared on equal grounds (see Stimuli section). Had we used a larger luminance difference between the lighter and the darker cues, we might have obtained a more pronounced saliency effect than with our *optimal* features, but the saliency would have not been comparable. Note that the saccadic latency advantage for the locations cued by high-luminance with respect to low-luminance features is comparable to that obtained by Kean et al. (2003)^31^.

*Optimal* and *non-optimal* features do not differ in low-level properties, such as average luminance or spatial frequency. Therefore, it is worthwhile to reflect upon the properties that makes *optimal* features so much more significant, to the point of eliciting the same effects as if they had different luminance. According to the reference model adopted here^13^, the visual system, to produce an early saliency map of a visual scene, extracts just a very limited set of *optimal* features*,* based on criteria of maximal entropy within strict bounds on data output rate. O*ptimal* features then represent a compromise between the amount of information they carry about the visual scene and the cost for the system to process them. On the other hand, *non-optimal* features are individually very informative, but do not meet computational limitations criteria.

Our findings confirm that the set of features identified by that reference model are indeed more salient than others also when used as implicit spatial cues in covert and overt attention tasks. The saliency map provided by these features seem thus to be used to automatically guide attention and eye movements towards informative locations, in addition to being used in the reconstruction of the image.

Interestingly, this suggests that computational limitations seem to take a significant role, not only in compression, but also in shaping what the visual system considers to be *salient.*

## Data Availability

The datasets generated and analyzed during the current study are available in the Zenodo repository (https://doi.org/10.5281/zenodo.5960548).
